# Beyond Mueller–Hinton agar: comparative evaluation of agar media for antibiotic susceptibility testing and implications for resource-limited laboratory settings

**DOI:** 10.1128/spectrum.04218-25

**Published:** 2026-05-29

**Authors:** Abraham Goodness Ogofure, Ezekiel Green, Etinosa Ogbomoede Igbinosa

**Affiliations:** 1 Department of Biotechnology and Food Technology, University of Johannesburg, Johannesburg, South Africa; 2 Microbial Pathogenicity and Molecular Epidemiology Research Group (MPMERG), Department of Biotechnology and Food Technology, University of Johannesburg, Johannesburg, South Africa; 3 Applied Microbial Processes and Environmental Health Research Group, Department of Microbiology, University of Benin 107745 , Benin, Nigeria; UCI Health, Orange, California, USA

**Keywords:** Mueller–Hinton agar, nutrient agar, Luria-Bertani agar, antimicrobial susceptibility testing, disk-diffusion assay

## Abstract

This study conducted a proof-of-concept risk assessment to compare the performance of available culture media with Mueller–Hinton agar (MHA) as the reference standard in antimicrobial susceptibility testing (AST) in resource-limited laboratories. Seven culture media were assessed using nine reference bacterial strains, tested against eight broad-spectrum antibiotics. Kirby–Bauer disc diffusion assay, zone diameter agreement (ZDA) of ±2 mm, similarity metrics, reproducibility indices, and multivariate approaches were performed. Data analysis was conducted in R-Studio using a comprehensive suite of statistical packages. Ward’s hierarchical clustering and Euclidean distance metrics revealed clear media-specific differences in antimicrobial diffusion patterns. MHA consistently produced the largest inhibition zones, approximately 75%, serving as the reference anchor for comparison. Others exhibited moderately comparable zone diameters, whereas nutrient and plate count agars produced consistently smaller zones. ZDA ranged from 66.7% (plate count agar [PCA]) to 81.9% (nutrient agar [NA] and peptone water agar [PWA]), with no medium achieving the ≥90% threshold used in published disk diffusion media comparisons. Categorical agreement assessed using CLSI breakpoints ranged from 92.3% to 96.2% per medium; no very major errors were observed, while major errors (2.6%) and minor errors (3.8%) were concentrated in two specific organism-antibiotic combinations, indicating a clinically relevant risk of interpretive error when MHA is substituted. Correlation analysis showed the strongest concordance for Luria-Bertani (r = 0.834) and rapid sensitivity test agar (r = 0.791). Most media underestimated zone diameters, but some demonstrated partial similarity to MHA, with none fully replicating its performance characteristics. These findings underscore the diagnostic risks associated with media substitution and highlight the need for strengthened AST.

**IMPORTANCE:**

Antimicrobial susceptibility testing (AST) is essential for guiding treatment of bacterial infections, yet its reliability depends on standardized media. In many resource-limited laboratories, Mueller–Hinton agar (MHA) is often substituted with more readily available media due to cost and access constraints. However, the performance and risks associated with this widespread practice remain poorly characterized. Our study provides critical data on the performance of commonly substituted media, including the magnitude and direction of interpretive errors when standard clinical breakpoints are applied. It also identifies specific organism-antibiotic combinations with the highest risk of misclassification. While some alternative media showed partial similarity, none fully replicated MHA’s performance. Our study does not advocate for modifying established standards or introducing new breakpoints, but documents a common practice, quantifies the associated diagnostic risks, and offers evidence-based guidance to reduce harm when standard methods are unavailable. These findings support quality improvement efforts and reinforce the need to strengthen diagnostic capacity in resource-limited settings.

## INTRODUCTION

One of the cornerstones of modern clinical microbiology is antimicrobial susceptibility testing (AST). Globally, AST provides essential guidance for therapeutic decision-making, antimicrobial stewardship programs, and the containment of antimicrobial resistance (AMR) (1–3). Among the recognized media for AST, Mueller–Hinton agar (MHA) is regarded as the gold standard by both the European Committee on Antimicrobial Susceptibility Testing (EUCAST) and the Clinical and Laboratory Standards Institute (CLSI) (4, 5). Its universal adoption is rooted in high reproducible performance, standardized composition, relative affordability, broad availability, and extensive validation across diverse bacterial pathogens. However, applying this standardization in resource-limited settings, such as in low- and middle-income countries (LMICs), presents considerable challenges, including inconsistent access to specialized media, quality control reagents, and standardized testing conditions, among other issues (6, 7).

Meanwhile, LMICs are disproportionately affected by the global burden of AMR, where restricted access to quality-assured testing materials, inadequate laboratory infrastructure, and limited diagnostic capacity contribute to the accelerated development of resistance and suboptimal patient outcomes (6–8). More often than not, in some of these (LMICs) settings, clinicians frequently resort to empirical antimicrobial therapy as a result of the unavailability or prohibitive cost of standardized AST (9, 10). This practice may lead to potential treatment failures and consequent increases in morbidity, mortality, and selection pressure for AMR pathogens. This gap in diagnostics poses a significant obstacle to effective antimicrobial stewardship and the implementation of precision medicine in regions where these interventions are most critically needed. In some resource-limited settings, implementing standardized AST protocols is often hindered by multiple challenges. The most notable among these is the irregular supply chain for specialized media. For example, MHA frequently experiences stock-outs lasting weeks or months in remote healthcare facilities. Furthermore, the requirement for strict storage conditions, including controlled humidity and temperature, complicates the logistics of media storage in tropical climates (1, 6, 8, 10). Moreover, when the relative cost of MHA preparation is compared to certain basic laboratory media, it becomes challenging for some healthcare facilities in LMICs operating under severe budgetary constraints to keep up.

In resource-limited settings, another significant hurdle is the quality control requirements, as the standard AST protocols mandate the use of specialized quality control strains (reference strains), which may be challenging to obtain and maintain, and have known susceptibility profiles (11, 12). The implementation of reliable AST is further compounded by the lack of trained personnel familiar with standardized AST interpretation criteria, as well as limited access to continuing education and proficiency testing programs.

Practically, some laboratories in LMICs routinely rely on general-purpose media, such as nutrient agar (NA), plate count agar (PCA), brain heart infusion agar (BHI), and Luria-Bertani agar (LBA), for organism recovery and enumeration (13–16). However, their performance in antimicrobial susceptibility testing, particularly under conditions of constrained resources, remains poorly studied or undocumented. This gap highlights a missed opportunity to critically evaluate whether readily available, nutritionally rich, and logistically accessible media can yield susceptibility results comparable to those obtained with Mueller–Hinton agar in resource-limited settings. Addressing this question is essential for evidence-based decision-making, whether to support local adaptation strategies or to justify stricter reliance on MHA. Most prior studies examine single organisms or antibiotics, but our study differs by integrating strain-level, antibiotic-specific, and multivariate analytical perspectives within a unified quantitative framework. We assessed nine reference strains of clinical and epidemiological relevance against a defined panel of broad-spectrum antibiotics on seven different media. By comparing inhibition zone diameters, error rates, categorical agreement, and interpretive consistency across media, the study seeks to determine whether any of these alternatives can reliably support antimicrobial susceptibility testing in resource-limited settings. Therefore, this study was designed as a proof-of-concept risk assessment to compare the performance of a panel of routinely available culture media with MHA as the reference standard in resource-limited laboratory settings. The study was not designed to determine whether alternative media can replace MHA; addressing that question would require a considerably larger study that incorporates a large and diverse cohort of clinical isolates. Accordingly, all findings from this study should be interpreted within the context of a comparative, proof-of-concept evaluation rather than as a basis for clinical implementation. More broadly, this work aims to provide evidence (for or against) that can inform context-appropriate AST practices and contribute to strengthening antimicrobial resistance surveillance capacity where access to standardized media is constrained. This study does not advocate replacing standardized Mueller–Hinton agar but rather evaluates the performance characteristics and risks of alternative media already used in resource-limited settings when standard materials are unavailable.

## MATERIALS AND METHODS

### Study design

The study was designed as a proof-of-concept risk assessment that characterizes the scope and magnitude of interpretive deviation, rather than a study designed to evaluate full media replacement. It was to assess whether routinely available culture media could yield results similar to those of Mueller–Hinton agar for antimicrobial susceptibility testing in resource-limited laboratories. Standard CLSI/EUCAST breakpoints are validated specifically for Mueller–Hinton agar. Application of these breakpoints to alternative media, as performed in this comparative study, is for evaluation purposes only and does not constitute a recommendation for clinical use. Comparative disc diffusion assays were performed on seven candidate media using nine reference bacterial strains and eight clinically significant antibiotics. Inhibition zone diameters, zone diameter agreement (ZDA), categorical agreement, and error profiles (very major, major, and minor errors) were evaluated to determine the suitability of each medium relative to MHA as the reference standard (17).

### Culture media selection and preparation

The seven culture media commonly used in diagnostic laboratories, which were evaluated, include Mueller–Hinton Agar (Oxoid, UK) as the reference medium, Rapid Sensitivity Test Agar (RSTA, TM Media, India), Brain Heart Infusion Agar (BHI, HiMedia, India), Nutrient Agar (NA, SRL product), Luria-Bertani Agar (LBA, Sigma-Aldrich, USA), Plate Count Agar (PCA, TM Media, India), and Peptone Water plus Bacteriological Agar (PWA, Merck and Oxoid combination). A non-nutrient saline agar prepared with bacteriological agar (Oxoid) was also included to test the limits of nutrient-poor and inefficient substrates. All the media were prepared according to the manufacturer’s instructions. Each was autoclaved at 121°C for 15 min, cooled to 45°C–50°C, and poured into sterile 90 mm Petri dishes at a uniform depth of 4 mm, in accordance with CLSI recommendations (18, 19). Sterility checks were performed by incubating uninoculated plates at 37°C for 24 h.

### Bacterial strains and culture

The study employed nine reference bacterial strains obtained from the American Type Culture Collection (ATCC) and the National Collection of Industrial, Food and Marine Bacteria (NCIMB), selected for their clinical and epidemiological relevance. These included *Staphylococcus aureus* ATCC 12493, *Bacillus subtilis* ATCC 11774, *Bacillus cereus* ATCC 33019, *Bacillus cereus* ATCC 10876, *Shigella sonnei* ATCC 29930, *Escherichia coli* ATCC 8739, *Enterococcus faecalis* ATCC 29212, *Lactobacillus acidophilus* ATCC 4356, and *Klebsiella aerogenes* NCIMB 10102. *L. acidophilus* was included to test media behavior with fastidious Gram-positive organisms rather than for direct clinical interpretability. All strains were revived on nutrient agar and incubated aerobically at 37°C for 18–24 h (20). Freshly grown colonies were used for each susceptibility assay.

### Inoculum standardization and antibiotic discs

Overnight agar cultures were suspended in sterile saline and adjusted to a 0.5 McFarland turbidity standard (approximately 1.5 × 10⁸ CFU per mL). A McFarland comparator (prepared by mixing 1% H_2_SO_4_ [99.5 mL] and 1.175% BaCl_2_ [0.5 mL]) was used as the standard, and the optical density of the solution was confirmed using a spectrophotometer. Inocula were applied within 15 min of preparation to prevent density drift and cultured on the plates. Eight broad-spectrum antibiotics covering major therapeutic classes were used in this assay. These included ofloxacin, amoxicillin–clavulanate, amikacin, clarithromycin, piperacillin–tazobactam, ceftriaxone, vancomycin, and meropenem (Oxoid, UK) (21, 22). Discs were stored under recommended conditions (refrigeration) and equilibrated to room temperature before testing.

### Kirby–Bauer disc diffusion susceptibility testing

Antimicrobial susceptibility testing was performed using the Kirby–Bauer disc diffusion method, adapted for comparative assessment across various media. Each plate was inoculated evenly using a sterile swab that was saturated with the standardized bacterial suspension. Antibiotic discs were placed aseptically at CLSI-recommended spacing. Plates were incubated at 37°C for 18–24 h, and each organism-antibiotic-medium combination was tested in replicates (*n =* 3) to ensure reproducibility (18). Each organism-antibiotic-medium combination was tested in triplicate across three independent experimental runs, permitting the calculation of standard errors, deviations, and coefficients of variation as measures of intra-medium reproducibility. Inhibition zones were measured in millimeters using a calibrated ruler. Irregular zones were measured along two perpendicular axes, and the mean value was recorded. Zone diameters obtained on MHA were interpreted according to CLSI (18) breakpoints. For non-MHA media, susceptibility categories were directly compared with MHA-derived results.

### Quality control validation

The nine reference bacterial strains used in this study were obtained from ATCC and NCIMB and were selected for their clinical and epidemiological relevance. Among them, *Enterococcus faecalis* ATCC 29212 is the sole designated CLSI disk diffusion quality control (QC) organism included in our panel. The remaining eight strains, *Staphylococcus aureus* ATCC 12493, *Bacillus subtilis* ATCC 11774, *Bacillus cereus* ATCC 33019, *Bacillus cereus* ATCC 10876, *Shigella sonnei* ATCC 29930, *Escherichia coli* ATCC 8739, *Lactobacillus acidophilus* ATCC 4356, and *Klebsiella aerogenes* NCIMB 10102, are well-characterized reference organisms but are not designated CLSI M100 disk diffusion QC strains; accordingly, formal CLSI QC pass/fail assessment using published zone diameter ranges cannot be applied to these organisms.

For *Enterococcus faecalis* ATCC 29212, CLSI M100 (18) publishes QC zone diameter ranges for a limited subset of antibiotics tested with this strain under standard disk diffusion conditions. Of the antibiotics included in our study, vancomycin has an established CLSI M100 QC range for *E. faecalis* ATCC 29212 of 17–21 mm. Quality control testing was performed in parallel with experimental assays under identical conditions using the Kirby–Bauer disk diffusion method on Mueller–Hinton agar, with replicate measurements obtained for all organism-antibiotic combinations. Although CLSI M100 provides QC zone diameter ranges for selected antibiotics tested against *E. faecalis* ATCC 29212, the observed zone diameters for the antibiotics evaluated in this study did not fall within the published QC ranges; therefore, formal QC pass/fail determination based on CLSI criteria could not be established. Accordingly, quality assurance was assessed based on internal consistency, including reproducibility across replicate measurements and the concordance of observed susceptibility patterns with established strain-specific phenotypes as documented in ATCC strain datasheets and the literature. All strains exhibited zone diameter patterns on Mueller–Hinton agar consistent with their expected phenotypic profiles, indicating that testing was conducted under appropriately standardized experimental conditions.

### Performance evaluation metrics

Performance metrics were evaluated using internationally recognized indicators to compare other evaluated media with MHA. It is worth noting that CLSI M100 breakpoints are method-specific and have been validated exclusively for use with Mueller–Hinton agar under standard disk diffusion conditions. Their application to zone diameters generated on non-MHA media in this study was performed solely to quantify and characterize the magnitude of potential interpretive deviation, and does not constitute a recommendation for clinical use of these breakpoints with evaluated media in the study. Primary metrics included ZDA, defined as the percentage of inhibition zone diameters on an alternative medium that fell within ±2 mm of the corresponding MHA value for the same organism-antibiotic combination, categorical agreement (CA), defined as the percentage of organism-antibiotic combinations for which the interpretive category (susceptible [S], intermediate [I], or resistant [R]) assigned on an alternative medium matched that assigned on MHA using CLSI M100 (2024) disk diffusion breakpoints, and error profiles comprising very major errors (VME: resistant on MHA, susceptible on alternative medium), major errors (ME: susceptible on MHA, resistant on alternative medium), and minor errors (mE: discrepancies involving the intermediate category in either the reference or test result) (18).

Categorical interpretations were assigned exclusively to organism-antibiotic combinations for which validated CLSI M100 disk diffusion breakpoints exist. Applicable organisms comprised *Staphylococcus aureus* ATCC 12493 (breakpoints applied for OFX, AMC, AK, TZP, CRO, and VA), *Enterococcus faecalis* ATCC 29212 (OFX and VA), *Escherichia coli* ATCC 8739, *Klebsiella aerogenes* NCIMB 10102, and *Shigella sonnei* ATCC 29930 (all as Enterobacterales: OFX, AMC, AK, TZP, CRO, and MEM). *Bacillus subtilis* ATCC 11774, *Bacillus cereus* ATCC 33019, *Bacillus cereus* ATCC 10876, and *Lactobacillus acidophilus* ATCC 4356 were excluded from categorical analyses because CLSI M100 does not provide validated disk diffusion breakpoints for these species. Clarithromycin (CLR) was similarly excluded from all CA analyses, as no CLSI M100 disk diffusion breakpoints are available for this antibiotic against any of the tested organisms. Vancomycin (VA) was excluded for all Gram-negative organisms (*E. coli, K. aerogenes, S. sonnei*) due to intrinsic resistance, and meropenem (MEM) was excluded for *S. aureus* and *E. faecalis* for the same reason. In total, 156 organism-antibiotic-medium comparisons across the six alternative media were evaluable for categorical agreement (18).

The CLSI M100 (2024) disk diffusion breakpoints applied are summarized as follows. For Enterobacterales: ofloxacin (S ≥ 18, I 15–17, R ≤ 14 mm), amoxicillin–clavulanate (S ≥ 18, I 14–17, R ≤ 13 mm), amikacin (S ≥ 17, I 15–16, R ≤ 14 mm), piperacillin–tazobactam (S ≥ 21, I 18–20, R ≤ 17 mm), ceftriaxone (S ≥ 23, I 20–22, R ≤ 19 mm), and meropenem (S ≥ 23, I 20–22, R ≤ 19 mm). For *Staphylococcus aureus*: ofloxacin (S ≥ 18, I 15–17, R ≤ 14 mm), amoxicillin–clavulanate (S ≥ 20, R ≤ 19 mm; no intermediate category), amikacin (S ≥ 17, I 15–16, R ≤ 14 mm), piperacillin–tazobactam (S ≥ 18, R ≤ 17 mm), ceftriaxone (S ≥ 18, R ≤ 17 mm), and vancomycin (S ≥ 15, I 12–14, R ≤ 11 mm). For *Enterococcus faecalis*: ofloxacin (S ≥ 16, I 13–15, R ≤ 12 mm) and vancomycin (S ≥ 17, I 15–16, R ≤ 14 mm). Additional indicators included pairwise similarity measures (Euclidean distance, mean absolute difference, and Pearson correlation coefficient), systematic bias quantified as the mean signed difference between alternative-medium and MHA zone diameters, paired statistical tests (paired *t*-test and Wilcoxon signed-rank test, selected by Shapiro–Wilk normality assessment), and reproducibility indices assessed as coefficients of variation (CV) across triplicate measurements for each organism-antibiotic-medium combination.

### Data visualization and statistical analysis

All data generated in this study were processed, statistically evaluated, and visualized using RStudio (version 4.5.0; 2025-04-11). Analytical workflows were implemented through a structured suite of R packages, including *tidyverse* for data wrangling, *psych* for descriptive statistics, *lme4* and *emmeans* for inferential modeling, *ggpubr* and *gridExtra* for publication-grade plotting, *FactoMineR* and *factoextra* for multivariate ordination, *TOSTER* and *pwr* for equivalence and power analyses, and *ComplexHeatmap* with *circlize* for advanced clustering and heatmap-based visual analytics. All libraries were programmatically installed and loaded to ensure reproducibility and consistency of the computational environment. The visualization strategy was deliberately aligned with analytical frameworks demonstrated in prior work (23–27), ensuring methodological continuity with established approaches for handling multidimensional antimicrobial susceptibility data sets. This included hierarchical clustering to profile media similarity, multivariate heatmaps to compare inhibition zone signatures, regression-based scatterplots to assess concordance with MHA, and ZDA to benchmark performance against accepted interpretive thresholds. It is worth noting that the non-nutrient saline agar was excluded from the final analyses due to its non-viability, as all strains struggled to grow on it.

## RESULTS

Prior to the comparative analysis, the quality of the experimental conditions was confirmed: the zone diameters obtained on MHA for all nine reference strains were consistent with their established susceptibility phenotypes. The mean vancomycin zone diameter obtained on MHA for *Enterococcus faecalis* ATCC 29212, the sole designated CLSI disk diffusion QC organism in the panel, was 7.5 mm, and the mean ofloxacin zone diameter was 9.5 mm. These values fall below the susceptible breakpoints for both agents according to CLSI M100 criteria (vancomycin: S ≥ 17 mm; ofloxacin: S ≥ 16 mm, I 13–15 mm) (18), classifying *E. faecalis* ATCC 29212 as resistant to both antibiotics in this study. This is consistent with the known reduced susceptibility of this strain to fluoroquinolones and its borderline-to-resistant profile to vancomycin, as documented in the literature (12). The observed phenotype on MHA is therefore consistent with the expected biological behavior of this reference strain. For the remaining eight reference strains, zone diameters obtained on MHA were compared with established susceptibility profiles documented in the published literature and ATCC strain datasheets. All strains produced zone diameter patterns consistent with their expected phenotypes: *S. aureus* ATCC 12493 demonstrated susceptibility to ofloxacin (24 mm) and amikacin (16 mm) and resistance to clarithromycin, meropenem, and piperacillin–tazobactam (0 mm) on MHA, consistent with documented profiles for non-MRSA *Staphylococcus aureus* with selective antibiotic resistances. Gram-negative organisms (*E. coli* ATCC 8739, *K. aerogenes* NCIMB 10102, *S. sonnei* ATCC 29930) showed expected intrinsic resistance patterns to vancomycin (0 mm) and variably reduced susceptibility to other agents. *B. subtilis* ATCC 11774 produced the largest zones overall, consistent with its broad susceptibility profile. These results confirm that testing was performed under valid and appropriately standardized conditions, and that observed inter-media differences in zone diameter reflect genuine medium-dependent variation in antibiotic diffusion rather than methodological artifacts.

**Fig 1:**
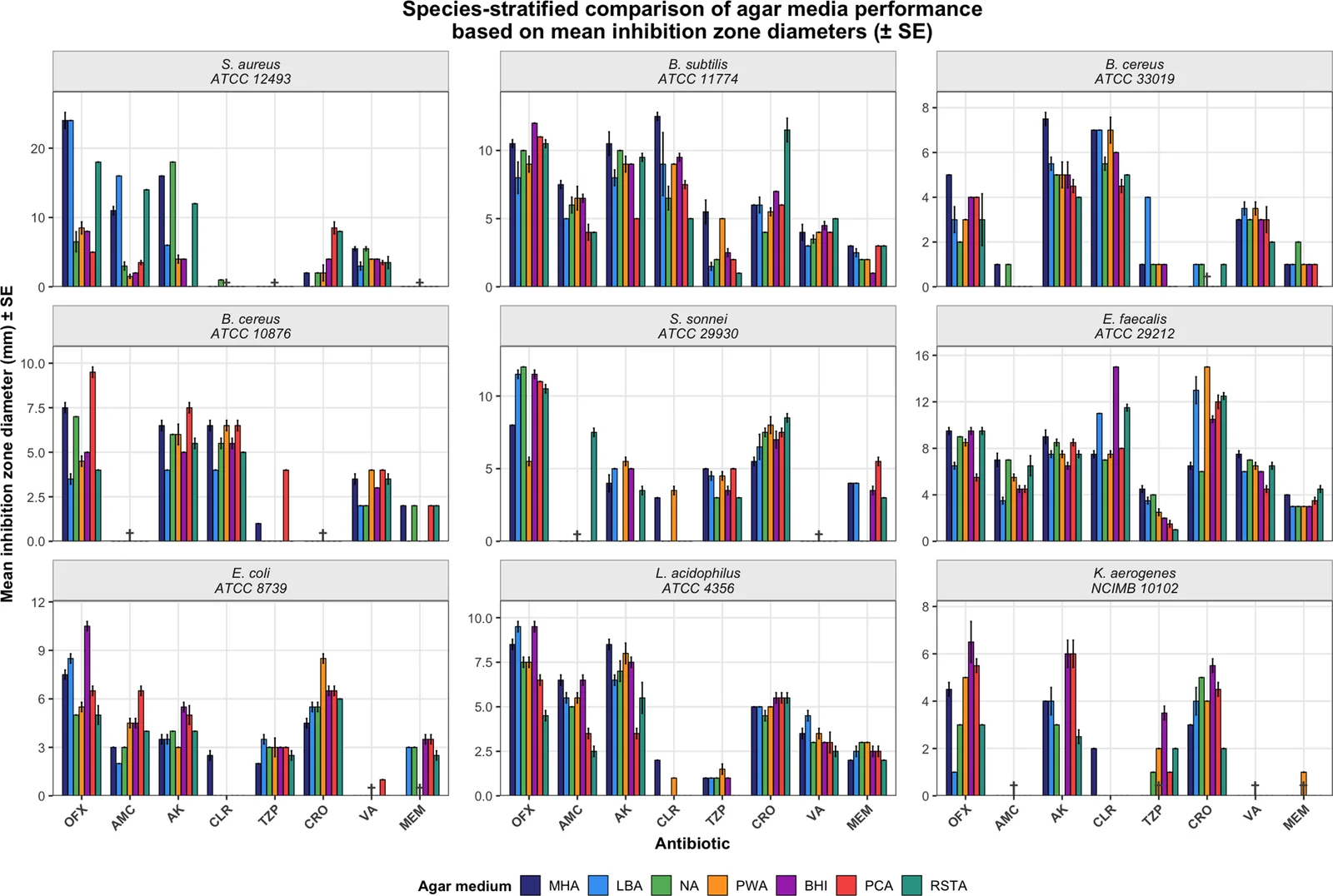
Species-stratified comparison of agar media performance based on mean inhibition zone diameters (±SE). Each of the nine panels corresponds to one reference bacterial strain tested in this study. Within each panel, the x-axis shows the eight antibiotics evaluated, and grouped bars represent the seven agar media (*n* = 3 replicates per organism-antibiotic-medium combination). The y-axis scale is free per panel to maximize readability across strains with different inhibition zone ranges; all panels derive from the same data set. The dagger symbol (†) marks organism-antibiotic combinations for which the MHA zone diameter = 0 mm, indicating intrinsic resistance or non-susceptibility irrespective of medium; these combinations were excluded from comparative performance calculations. The original cross-media heatmap aggregated across all strains is provided as Fig. S1 for descriptive reference. Antibiotic abbreviations: OFX, ofloxacin; AMC, amoxicillin–clavulanate; AK, amikacin; CLR, clarithromycin; TZP, piperacillin–tazobactam; CRO, ceftriaxone; VA, vancomycin; MEM, meropenem. Media abbreviations: MHA, Mueller–Hinton agar; LBA, Luria-Bertani agar; NA, nutrient agar; PWA, peptone water agar; BHI, brain heart infusion agar; PCA, plate count agar; RSTA, rapid sensitivity test agar.

The comparative performance of seven culture media (MHA, RSTA, BHI, LBA, NA, PWA, and PCA) on the species-stratified comparison of agar media performance based on mean (±SE) inhibition zone diameters is shown in Fig. 1. Each of the nine panels displays mean inhibition zone diameters (±SE) for all seven evaluated media across eight antibiotics, enabling biologically valid within-organism comparisons of media performance. Organism-antibiotic combinations for which the MHA zone diameter equals zero (indicating intrinsic resistance or non-susceptibility irrespective of the medium used) are flagged with a dagger (†) sign and excluded from comparative analyses; a total of 14 such combinations were identified across the nine strains. For organisms with well-characterized susceptibility profiles, clear medium-dependent differences were apparent. In the *S. aureus* panel, MHA produced the largest zone for OFX (mean 24 mm), with BHI, NA, PWA, and PCA yielding markedly smaller zones (4–8.5 mm), a pattern that directly contributes to the major interpretive errors (false-resistant classifications) reported in Table 1. Similarly, in the *E. faecalis* and Enterobacterales panels (*E. coli, K. aerogenes, S. sonnei*), differences between MHA and alternative evaluated media are antibiotic-specific and variable rather than uniform, emphasizing that no single alternative medium consistently approximates MHA across all organism-antibiotic combinations. Strains producing uniformly small zones regardless of medium (including *K. aerogenes* [max mean MHA zone 4.5 mm] and *B. cereus* strains) demonstrate that the observed inter-media differences for these organisms are relatively small in absolute terms, though their potential to shift categorical classifications near breakpoint boundaries remains clinically relevant. Collectively, Fig. 1 shows that media effects on zone diameter are both organism- and antibiotic-specific, reinforcing the idea that no single alternative medium reliably reproduces MHA performance across the full breadth of tested organism-antibiotic combinations. A look at the mean inhibition zone diameters generated across nine reference bacterial strains and eight broad-spectrum antibiotics (shown in Fig. S1 to S3 using the Ward’s method for hierarchical clustering, Euclidean distance metrics, and heatmap) revealed a clear media-specific variation in antimicrobial diffusion and inhibitory response patterns relative to MHA. Unsurprisingly, MHA consistently produced the largest zones for about 75% of the tested antibiotics, serving as the reference anchor for both clustering and statistical comparisons. The largest average inhibition zones produced by MHA reflect its optimized formulation for antimicrobial diffusion and its established role as the reference standard for disk diffusion AST. Among the evaluated media in the study, RSTA, BHI, and LBA demonstrated moderately high mean zone diameters relative to MHA, whereas NA, PWA, and PCA produced consistently smaller mean zone diameters (Fig. S1). These differences indicate variable antimicrobial diffusion efficiencies across media, likely reflecting differences in nutrient composition, ionic content, and gel matrix properties. Therefore, media, such as RSTA, BHI, and LBA, were grouped proximally to MHA, reflecting broadly comparable zone distributions, whereas NA, PWA, and PCA exhibited progressively lower inhibition profiles, indicating reduced antimicrobial diffusion or altered matrix-drug interactions. The accompanying right-side annotations (in Fig. S3) summarized two critical metrics, which are statistical concordance (*P <* 0.05), highlighting which media differ significantly from MHA in paired comparisons, and the Euclidean distance from MHA, quantifying how closely each medium approximates the inhibition profile of the standard (Fig. S2). It should be noted that larger average zone diameters do not necessarily imply better suitability for AST, as interpretive accuracy depends on categorical agreement, error profiles, and consistency relative to MHA, and not on absolute zone magnitude alone. The figure, therefore, provides an initial overview of media-dependent differences in inhibitory performance, serving as a precursor to the deeper comparative analyses presented in subsequent figures. Collectively, these figures (Fig. 1; Fig. S1 to S4) demonstrate that although several routinely accessible media display partial similarity to MHA, none fully replicate its diffusion and performance characteristics across all antibiotic classes evaluated. This finding underscores the need for caution when substituting MHA in resource-limited laboratories and provides empirical grounding for evaluating which alternative agars (if any) show promise for reliable AST when MHA is limited in supply.

**TABLE 1:** CA and error rates for six alternative culture media relative to MHA as the reference standard, based on CLSI M100 (2024) disk diffusion breakpoints^*a*^

Medium	Applicable combinations (*n*)	Categorical agreement (CA) *n* (%)	Very major errors (VME) *n* (%)	Major errors (ME) *n* (%)	Minor errors (mE) *n* (%)	Zone diameter agreement (%) (±2 mm vs MHA)
LBA	26	25 (96.20)	0 (0.00)	0 (0.00)	1 (3.80)	70.8
RSTA	26	25 (96.20)	0 (0.00)	0 (0.00)	1 (3.80)	68.1
BHI	26	24 (92.30)	0 (0.00)	1 (3.80)	1 (3.80)	73.6
NA	26	24 (92.30)	0 (0.00)	1 (3.80)	1 (3.80)	81.9
PWA	26	24 (92.30)	0 (0.00)	1 (3.80)	1 (3.80)	81.9
PCA	26	24 (92.30)	0 (0.00)	1 (3.80)	1 (3.80)	66.7
Summary	156	146 (93.60)	0 (0.00)	4 (2.60)	6 (3.80)	73.8

^*a*^ Key: applicable combinations are those for which validated CLSI M100 disk diffusion breakpoints exist for the tested organism-antibiotic pair. *Bacillus* spp., *Lactobacillus acidophilus*, and clarithromycin were excluded from categorical analyses due to the absence of CLSI breakpoints. Evaluable combinations comprised five bacterial species (*Staphylococcus aureus, Enterococcus faecalis, Escherichia coli, Klebsiella aerogenes, Shigella sonnei*) and six antibiotics (OFX, AMC, AK, TZP, CRO, VA, and/or MEM) per medium (*n* = 26 per medium; 156 total). VME, very major error (resistant on MHA, susceptible on alternative medium). ME, major error (susceptible on MHA, resistant on alternative medium). mE, minor error (discrepancy involving the intermediate category). ZDA, zone diameter agreement (percentage of all 72 organism-antibiotic combinations per medium with zone diameter within ±2 mm of MHA). MHA, Mueller–Hinton agar; LBA, Luria-Bertani agar; NA, nutrient agar; PWA, peptone water agar; BHI, brain heart infusion agar; PCA, plate count agar; RSTA, rapid sensitivity test agar.

The patterns of interaction between all nine reference bacterial strains and the seven evaluated media, based on mean inhibition zone diameters (Fig. S5), highlight clear heterogeneity in antimicrobial diffusion patterns across strain-medium combinations, with both absolute zone sizes and relative response profiles contributing to the clustering structure. It should be noted that Fig. S5 presents a hierarchical clustering heatmap based on zone-diameter averages across antibiotic classes and is provided for descriptive purposes only; due to this averaging, it should not be used to infer media performance for specific organism-antibiotic combinations*.* While MHA generally produced larger zones of inhibition across most bacterial strains, other evaluated media showed variable, strain-dependent performance rather than a uniform deviation from the reference MHA. The column dendrogram indicates that other media cluster primarily by similarity in response patterns across strains, rather than solely by the magnitude of inhibition zones, underscoring that media with smaller zones may still share comparable strain-specific trends. The row dendrogram reveals distinct groupings of bacterial strains based on their overall inhibition zone diameter profiles across media. Strains, such as *Enterococcus faecalis, Bacillus subtilis*, and *Staphylococcus aureus,* exhibit higher mean inhibition responses and cluster together, whereas *Klebsiella aerogenes*, *Bacillus cereus* strains, and *Escherichia coli* form separate clusters characterized by lower or more variable zone diameters. Collectively, these patterns demonstrate that both agar composition and bacterial species exert strong and interacting effects on inhibition zone outcomes, highlighting the strain-specific nature of performance deviations observed when non-standard media are used for AST. These patterns underscore the challenge of substituting MHA for non-standard media, as performance deviations are strain-dependent and medium-specific, with potential consequences for interpretive accuracy and AST reliability in resource-limited settings.

**Fig 2:**
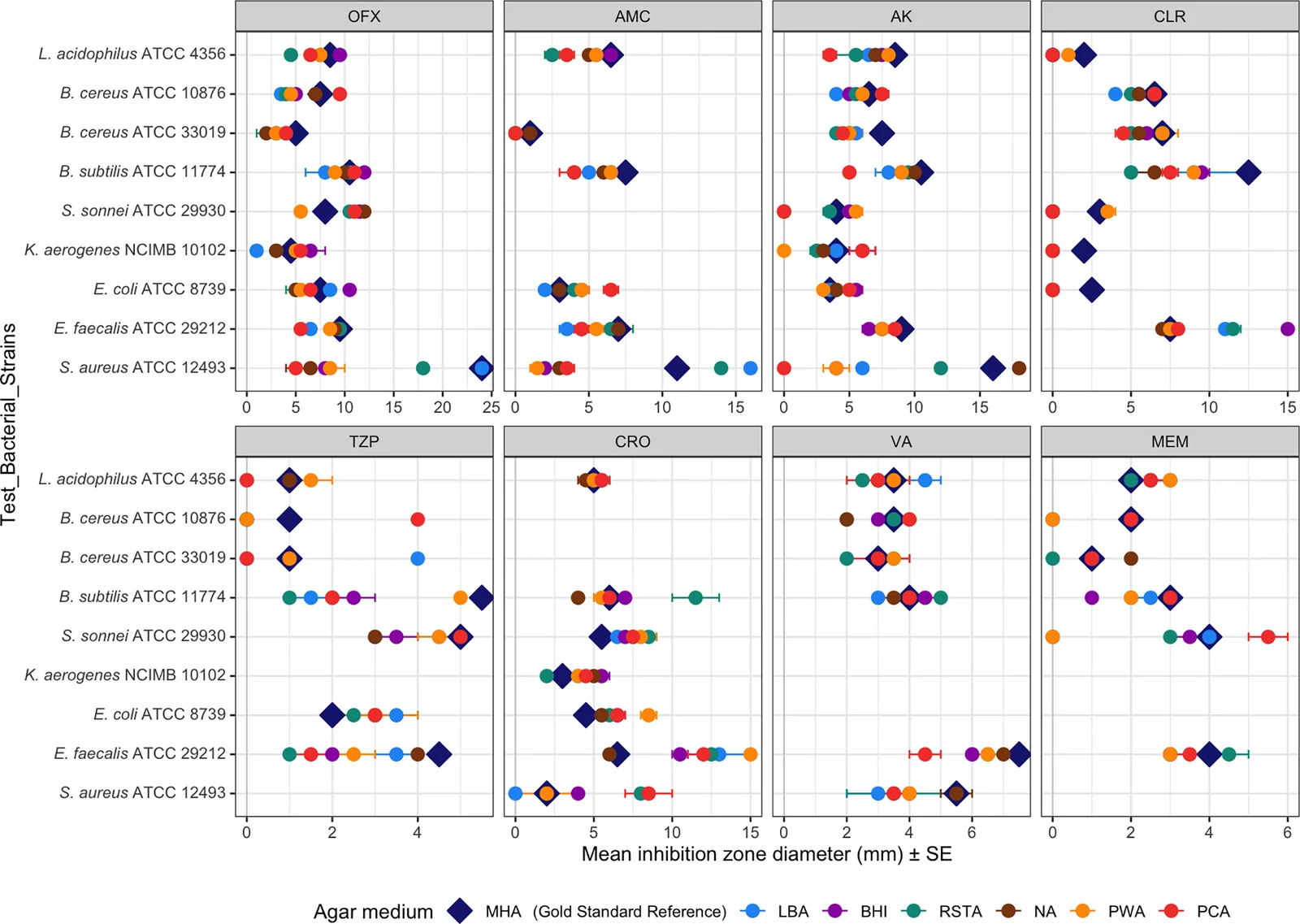
Forest-style plot of mean inhibition zone diameters by organism type within each antibiotic group. Key: antibiotic abbreviations: OFX, ofloxacin; AMC, amoxicillin–clavulanate; AK, amikacin; CLR, clarithromycin; TZP, piperacillin–tazobactam; CRO, ceftriaxone; VA, vancomycin; and MEM, meropenem.

A forest-style visualization of mean inhibition zone diameters (mm ± SE) for each organism-antibiotic combination across the evaluated agar media, stratified by antibiotic class, is presented in Fig. 2. Across most antibiotic groups, Mueller–Hinton agar (MHA; reference standard) produced the largest inhibition zones for the majority of organism-antibiotic combinations, consistent with its optimized formulation for antimicrobial diffusion. However, this pattern was not uniform across all conditions, and deviations were observed depending on the organism-antibiotic context. The evaluated alternative media exhibited variable and organism-dependent differences relative to MHA, with no single medium maintaining consistent agreement across all conditions. Media, such as LBA, BHI, and RSTA, showed relatively closer agreement with MHA for selected antibiotics (notably OFX and AK), whereas NA, PWA, and PCA generally produced smaller inhibition zone diameters, suggesting differences in antimicrobial diffusion behavior or medium-drug interactions. Importantly, the magnitude and direction of deviation varied across both organisms and antibiotics. For example, more pronounced discrepancies were observed for *Staphylococcus aureus* and *Enterococcus faecalis* in the fluoroquinolone (OFX) and aminoglycoside (AK) panels, whereas more uniform and compressed zone distributions were observed for glycopeptide (VA) and carbapenem (MEM) panels, reflecting reduced variability across media for these agents.

Variation in the extent of overlap among mean ± SE intervals across organism-antibiotic combinations indicates differing levels of agreement between MHA and other evaluated media.

These findings demonstrate that media-dependent effects on inhibition zone diameters are highly organism-specific and antibiotic-specific, reinforcing that antimicrobial susceptibility testing results derived from non-standard media cannot be generalized across species or drug classes.

**Fig 3:**
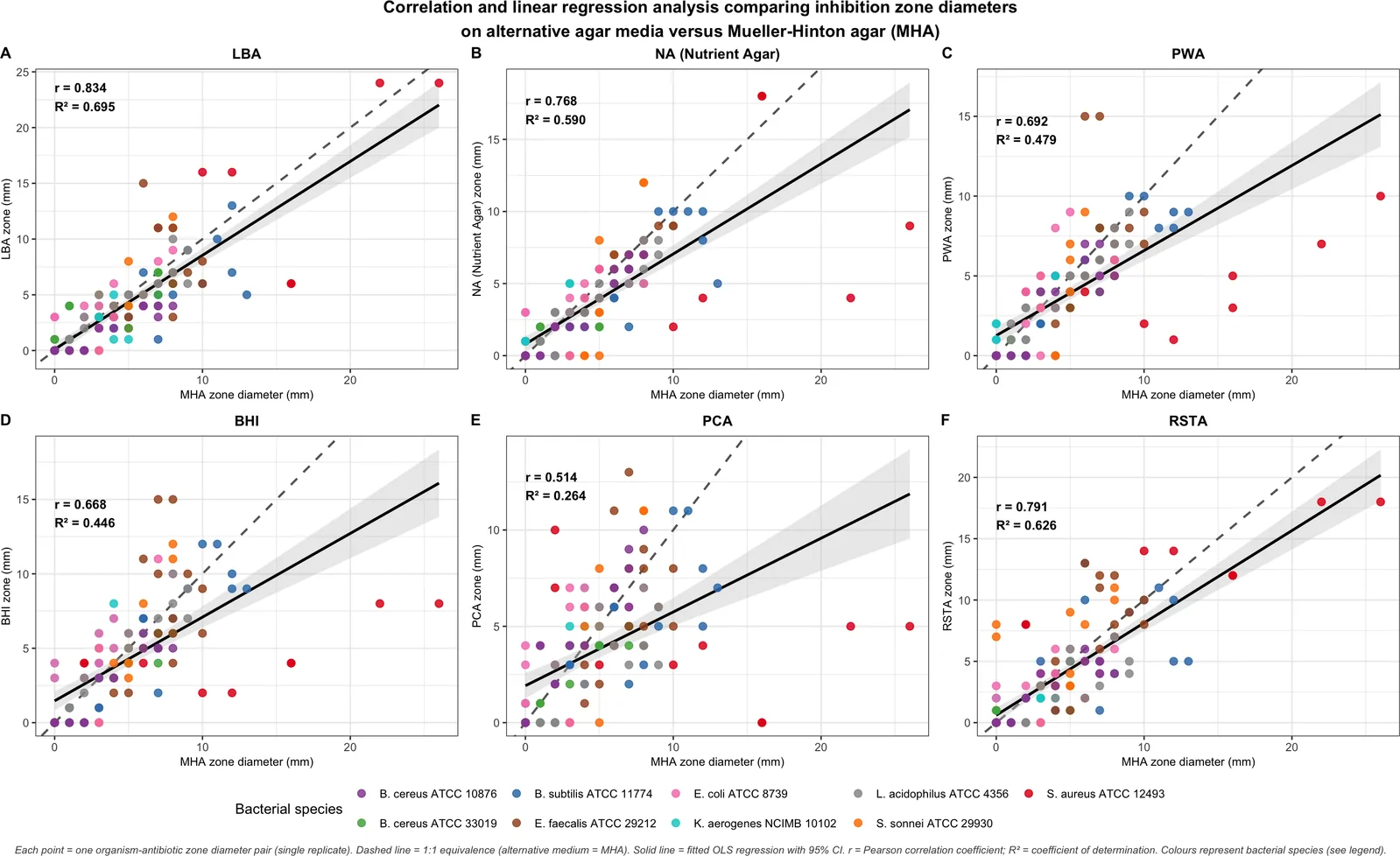
Correlation and linear regression analysis comparing inhibition zone diameters on alternative agar media versus MHA.

To determine whether the observed inter-media differences in inhibition zone diameters were statistically significant, pairwise comparisons between each alternative medium and MHA were performed for each antibiotic panel, with *n* = 27 paired measurements (nine reference strains × three replicates). For each comparison (Fig. S6), the Shapiro-Wilk test was applied to the distribution of pairwise differences to assess normality; where normality was supported (*P* > 0.05), a paired *t*-test was used, and where it was violated (*P* ≤ 0.05), the Wilcoxon signed-rank test was applied. Significance annotations are displayed directly on Fig. 3 above each alternative medium bar, and the full statistical output is provided in Table S1. Statistically significant reductions relative to MHA were most pronounced for OFX (PWA: *P* = 0.0009***; RSTA: *P* = 0.0077**), AK (RSTA: *P* = 0.0005***; LBA: *P* = 0.0066**; PWA: *P* = 0.0119*; PCA: *P* = 0.0183*), and CLR (PCA: *P* = 0.0004***; NA: *P* = 0.0009***; LBA, PWA, and RSTA: *P* < 0.05*). In contrast, for CRO, several alternative media (PWA, BHI, PCA, and RSTA) produced zone diameters significantly larger than MHA (*P* < 0.05 to *P* < 0.01), reflecting a positive bias for this antibiotic on these media. For TZP, VA, and MEM, differences versus MHA were largely non-significant across all alternative media, consistent with the generally small zone diameters observed for these agents regardless of medium. Collectively, these results confirm that the inter-media zone diameter differences shown in Fig. S6 are not merely numerical artifacts but reflect real, medium-specific effects on antibiotic diffusion that are statistically detectable for the more differentiating antibiotics (OFX, AK, CLR) and absent for agents generating uniformly small zones across all media (TZP, VA, MEM).

The correlation and linear regression analysis comparing inhibition zone diameters on alternative agar media versus MHA is shown in Fig. 3. The scatterplots with linear regression models comparing inhibition zone diameters obtained on the six evaluated media (LBA, NA, PWA, BHI, PCA, and RSTA) with MHA showed paired zone measurements across all strain-antibiotic combinations. The results further highlight the quantitative agreement, proportional bias, and overall comparability with the other media evaluated. LBA and RSTA demonstrated the strongest correlations with MHA (r = 0.834 and r = 0.791, respectively), with corresponding R² values (0.695 and 0.626) indicating substantial shared variance and relatively consistent proportional performance across the tested antimicrobial agents. NA and BHI showed moderate correlations (r = 0.768 and r = 0.668), though greater dispersion around the regression line suggests increased variability in antibiotic diffusion. PWA exhibited a similar moderate association (r = 0.692, R² = 0.479), whereas PCA showed the weakest correlation (r = 0.514, R² = 0.264), reflecting poor predictive power and inconsistent agreement with MHA. It can also be observed that, across all panels (Fig. 3), the regression slopes fall below the ideal equivalence (the 1:1 dashed line), indicating that the alternative media systematically underestimate inhibition zones. The extent of deviation varies by medium, with PCA and BHI showing the most pronounced divergence. These regression analyses emphasize that while some readily available media approximate MHA performance to a limited degree, none achieve the reproducibility or quantitative fidelity required for reliable antimicrobial susceptibility testing. Our findings thereby reinforce the need for caution when substituting MHA in resource-limited settings, as variability in diffusion characteristics may compromise interpretive accuracy.

The ZDA between inhibition zone diameters measured on six alternative agar media and the MHA reference standard, using a ±2 mm acceptability threshold, is shown in Table 1. The ZDA represents the proportion of organism-antibiotic combinations for which a medium’s zone diameter falls within ±2 mm of MHA. A dashed vertical line at the 90% threshold is shown for reference; this threshold is applied pragmatically in published disk diffusion media comparisons (4, 28) and is not a formal CLSI requirement for disk diffusion. None of the alternative agar media achieved a ZDA of 90% or higher within ±2 mm of MHA. NA and PWA demonstrated the highest ZDA (81.9% each), followed by BHI (73.6%) and LBA (70.8%). RSTA (68.1%) and PCA (66.7%) showed the lowest agreement in zone diameter.

The descending pattern of ZDA reinforces that these general-purpose or nutrient-rich media alter inhibition zone expression sufficiently to fall outside acceptable equivalence margins, even when they maintain predictable susceptibility trends. These deviations, though sometimes look minimal, carry severe clinical importance because discrepancies beyond ±2 mm can shift categorical interpretations, especially near breakpoint thresholds. It should be noted that while several media come moderately close to MHA, none provide the level of accuracy required for reliable antimicrobial susceptibility testing, particularly in resource-limited settings where diagnostic errors carry substantial consequences for patient management and AMR surveillance.

Bias analysis confirmed a consistent negative bias across all alternatives, with mean differences ranging from −0.52 to −0.91 mm relative to MHA and statistically significant *P* values for all media (Table S2). Together with the ZDA and correlation results, this suggests that alternative agars tend to yield slightly smaller zones than MHA but do so in a relatively systematic way. When the different performance dimensions were integrated into a composite ranking that weighted Euclidean distance (40%), zone diameter agreement (25%), correlation (25%), reproducibility (5%), and bias (5%), LBA and BHI emerged as the most MHA-like medium (composite score ≈60–64/100), then followed by RSTA and NA (≈60/100). PWA occupied an intermediate tier, whereas PCA ranked last (≈38–50/100) due to its greater distance from MHA, weaker correlation, lower ZDA, and higher variability (Fig. S5). For laboratories in resource-limited settings that cannot reliably procure MHA, these data indicate that LBA and BHI, and to a lesser extent NA, performed better than the other evaluated media. However, none of the tested media achieved equivalence to MHA, and their use for antimicrobial susceptibility testing should be interpreted with caution and limited to context-specific, non-clinical, or exploratory applications.

Furthermore, reproducibility, as measured by CV, was acceptable across media and broadly comparable to that of MHA. However, NA showed the lowest mean CV (approximately 5%), suggesting highly consistent measurements and thus good within-medium precision. BHI and MHA themselves had mean CVs around 8%, while RSTA, PCA, and LBA exhibited slightly higher dispersion (11%–14%), and PWA showed the highest variability (approximately 15%). These patterns indicate that general-purpose media, particularly NA and BHI, can support reproducible measurements, even if their absolute zone diameters differ modestly from those on MHA.

To evaluate the clinical interpretive implications of using alternative media in place of MHA, CA, and error rates were assessed for all organism-antibiotic-medium combinations to which validated CLSI M100 (2024) disk diffusion breakpoints were applicable. A total of 156 evaluable comparisons were generated across the six media (26 per medium), drawn from five organisms (*S. aureus, E. faecalis, E. coli, K. aerogenes,* and *S. sonnei*) and six antibiotics (OFX, AMC, AK, TZP, CRO, VA, and MEM; CLR was excluded as detailed in the Materials and Methods). The results are summarized in Table 1 (per medium) and Table 2 (per antibiotic). We categorically emphasize that the CLSI M100 breakpoints were applied to non-MHA zone diameters solely to evaluate media-induced interpretive error, and that this approach does not constitute a recommendation for routine clinical application. Generally, the categorical agreement across all six alternative media was 93.6% (146/156 evaluable combinations). No very major errors (VME; false-susceptible classification of a resistant organism) were detected for any medium (VME rate: 0.0% across all 156 comparisons). Four major errors (ME; false-resistant classification of a susceptible organism) were identified, yielding an overall ME rate of 2.6%, and six minor errors (mE; discrepancies involving the intermediate category) were recorded, giving a mE rate of 3.8%. When stratified by medium (Table 1), LBA and RSTA demonstrated the highest categorical agreement at 96.2% each (25/26 applicable comparisons), with only one mE per medium and no VMEs or MEs. These two media also achieved 100% CA for ofloxacin against *S. aureus*, the antibiotic-organism pair generating the most interpretive errors in other media. BHI, NA, PWA, and PCA each recorded CA rates of 92.3% (24/26), all with one ME and one mE, and no VMEs. The ME in each of these four media arose from the same organism-antibiotic combination, ofloxacin tested against *S. aureus* ATCC 12493, where the MHA zone diameter of 24 mm classified the organism as susceptible (S ≥ 18 mm by CLSI), while BHI, NA, PWA, and PCA generated considerably smaller zone diameters (4.0–8.5 mm), leading to false-resistant classification. This finding is consistent with the pronounced antibiotic-specific and medium-specific diffusion differences observed in Fig. 1 and 3 for ofloxacin across these media.

**TABLE 2:** CA and error rates for eight antibiotics across all six alternative media combined, relative to MHA, calculated using CLSI M100 (2024) disk diffusion breakpoints^*a*^

Antibiotic	Applicable combinations (*n*)	Categorical agreement (CA) *n* (%)	Very major errors (VME) *n* (%)	Major errors (ME) *n* (%)	Minor errors (mE) *n* (%)
OFX (ofloxacin)	30	26 (86.70)	0 (0.00)	4 (13.30)	0 (0.00)
AMC (amoxicillin–clavulanate)	24	24 (100.00)	0 (0.00)	0 (0.00)	0 (0.00)
AK (amikacin)	24	18 (75.00)	0 (0.00)	0 (0.00)	6 (25.00)
CLR (clarithromycin)	–	–	–	–	–
TZP (piperacillin–tazobactam)	24	24 (100.00)	0 (0.00)	0 (0.00)	0 (0.00)
CRO (ceftriaxone)	24	24 (100.00)	0 (0.00)	0 (0.00)	0 (0.00)
VA (vancomycin)	12	12 (100.00)	0 (0.00)	0 (0.00)	0 (0.00)
MEM (meropenem)	18	18 (100.00)	0 (0.00)	0 (0.00)	0 (0.00)
Overall	156	146 (93.60)	0 (0.00)	4 (2.60)	6 (3.80)

^*a*^ Key: *n* = 0 and all metrics = N/A for CLR as no CLSI M100 disk diffusion breakpoints exist for clarithromycin against the tested organisms. “–” denotes not applicable*. *

The mE observed in each medium was also attributable to a single organism-antibiotic combination: amikacin tested against *S. aureus* ATCC 12493, with an MHA zone diameter of 16 mm, which fell in the intermediate category (I: 15–16 mm by CLSI). Alternative media generated both downward (BHI 4.0 mm, LBA 6.0 mm, PCA 0 mm, PWA 4.0 mm, RSTA 12.0 mm → reclassified as R) and upward (NA 18.0 mm → reclassified as S) shifts, underscoring the particular interpretive vulnerability of isolates with zone diameters close to breakpoint boundaries when non-standard media are employed.

When stratified by antibiotic (Table 2), OFX exhibited the lowest CA rate at 86.7% (26/30), driven by the four MEs described above across BHI, NA, PWA, and PCA for *S. aureus*. AK showed a CA rate of 75.0% (18/24), with all six discordances classified as mEs, reflecting medium-induced shifts across the narrow 15–16 mm intermediate zone. AMC, TZP, CRO, VA, and MEM all achieved 100% CA across all applicable media and organisms (Table 2), indicating that media-induced differences in zone diameter for these agents did not result in interpretive category changes relative to MHA under the tested conditions. These high-CA antibiotics are predominantly those for which all tested organisms were classified as resistant on MHA, and alternative media similarly failed to reach susceptibility thresholds, limiting the scope for category discordance. Zone diameter agreement (ZDA, ±2 mm) per medium ranged from 66.7% (PCA) to 81.9% (NA and PWA), with LBA at 70.8%, RSTA at 68.1%, and BHI at 73.6% (Table 1). The lower ZDA rates compared with CA rates reflect the high sensitivity of the ±2 mm threshold relative to the coarser S/I/R categorization; many zone diameter differences that exceed 2 mm do not cross a categorical breakpoint boundary, particularly in combinations where all media and MHA agree on resistance. These findings collectively demonstrate that while alternative media introduce quantitative zone diameter differences that frequently exceed the ±2 mm ZDA threshold, the resultant categorical interpretive error rate is substantially lower, concentrated in two specific organism-antibiotic combinations (OFX/*S. aureus* and AK/*S. aureus*), and dominated by MEs and mEs rather than the more clinically serious VME class. No VMEs were generated by any medium, indicating that none of the tested alternative media caused a false-susceptible result for an organism classified as resistant on MHA within the evaluable combinations.

The total number of applicable comparisons per antibiotic reflects the number of evaluable organism-medium pairs across all six alternative media. CLR (clarithromycin) had no applicable CLSI M100 disk diffusion breakpoints for any tested organism and was therefore excluded from categorical analysis. Antibiotic abbreviations: OFX, ofloxacin; AMC, amoxicillin–clavulanate; AK, amikacin; CLR, clarithromycin; TZP, piperacillin–tazobactam; CRO, ceftriaxone; VA, vancomycin; MEM, meropenem. Error type definitions are as in Table 1.

## DISCUSSION

This study was conducted as a proof-of-concept risk assessment, and the results should be interpreted accordingly. CLSI M100 interpretive breakpoints are method-specific and validated exclusively for Mueller–Hinton agar; therefore, their application to zone diameters generated on non-MHA media in this study was undertaken solely to quantify potential interpretive deviation. Consequently, these findings should be interpreted as comparative performance data and must not be used for clinical susceptibility categorization on non-MHA media. Importantly, the limited isolate panel (*n* = 9) used in this proof-of-concept study restricts the generalizability of these findings across organisms, resistance phenotypes, and resistance mechanisms. A considerably larger validation study incorporating a diverse cohort of clinical isolates would be necessary to robustly evaluate whether any of the evaluated media can perform reliably or approximate the performance of MHA for routine AST. The study demonstrates that although several readily available culture media can approximate the behavior of MHA, none can fully reproduce its diffusion characteristics or interpretive performance across the diverse organism-antibiotic panel evaluated. MHA consistently produced the largest inhibition zones for most antibiotic-strain combinations and served as the central node in hierarchical clustering, which aligns with its carefully optimized composition, cation content, and agar concentration for disk-diffusion testing in international guidelines. Multiple studies confirm that the composition and physical properties of culture media, such as nutrient content, ionic strength, and gel matrix, significantly influence antibiotic diffusion and the size of the inhibition zone (20, 28, 29). For example, in our study, NA produces smaller, more variable inhibition zones than MHA, potentially leading to high error rates and unreliable susceptibility results, especially for certain bacterial species and antibiotics. This unreliability is attributed to differences in agar composition, which affect both antibiotic diffusion and bacterial growth. Other studies have shown that even among commercial MHA brands, differences in cation content and pH can result in significant variability in inhibition zone diameters, particularly for specific antibiotic classes (4, 20, 28). This underscores why any departure from MHA in routine AST must be approached cautiously. The other media evaluated in our study showed a spectrum of similarity to MHA rather than a binary pattern of equivalence or non-equivalence. These formulations, such as BHI, LBA, and RSTA, clustered proximally to MHA and produced moderately high mean zone diameters, with comparatively low Euclidean distances and mean absolute differences, indicating broadly similar inhibition profiles. These findings are concordant with smaller evaluations in which richer media sometimes yield zone sizes close to MHA, particularly for fastidious organisms or certain β-lactams (29, 30). By contrast, PWA and PCA consistently produced smaller zones and clustered farther from the reference, consistent with prior observations that non-standard or food-grade media can dampen antibiotic diffusion and alter zone morphology due to differences in agar strength, nutrient composition, and buffering capacity. Importantly, our study extends from previous single-species or single-drug reports (17, 31) by systematically quantifying these deviations across nine reference strains and eight broad-spectrum agents, providing a more generalizable picture of media-dependent variability.

Although some of the evaluated media, such as BHI, LBA, and RSTA, had mean inhibition zone diameters close to that of MHA, none of them consistently achieve the critical benchmark of ≥90% ZDA threshold within ±2 mm of MHA, and all display statistically significant negative biases, typically underestimating inhibition zones by 0.5–0.9 mm. Several studies evaluating alternative and enriched media for AST consistently report that these media fall short of the ≥90% ZDA threshold used in published disk diffusion comparisons (20, 28, 29, 32). Even when mean zone diameters are close, the proportion of results within ±2 mm of MHA is insufficient for clinical reliability (28, 29, 32). Even though the standardized mean differences (Cohen’s d) in our study fell within the small-effect range, a modest systematic shift of around 1 mm can affect categorical interpretation close to clinical breakpoints. This has been shown in experimental studies manipulating agar depth or composition within nominally standard MHA (2, 4, 28). The divergence between parametric and non-parametric paired tests for BHI further suggests that some media exhibit non-normal difference distributions, reinforcing the need to probe assumptions when judging equivalence. Reproducibility was generally acceptable across media, with coefficients of variation comparable to or only slightly higher than those observed for MHA, echoing reports that precision can remain high even when absolute zone sizes drift due to subtle compositional differences. These findings confirm that one of the key limitations of alternative media in our data set is not random noise but systematic, medium-dependent bias relative to MHA. Therefore, from a health-systems perspective, these results are particularly relevant for laboratories in LMICs, where constrained budgets and unstable supply chains often necessitate the use of whatever media are available rather than ideal formulations. Surveys and reviews of clinical bacteriology in LMICs document frequent reliance on NA, in-house media, or general-purpose agar due to cost and procurement challenges, with limited validation of their suitability for AST (13, 15, 20). Our present data support and refine these concerns: NA and PWA, while more accessible, showed substantial deviations from MHA in ZDA and bias, and PCA performed worst across nearly all metrics. Simultaneously, the partial convergence of BHI, LBA, and RSTA with MHA indicates that these media demonstrated relatively closer agreement with MHA than the other evaluated media. However, none achieved equivalence to MHA, and their use as substitutes for antimicrobial susceptibility testing cannot be recommended based on the present data set alone; the results must therefore be interpreted with caution. This perspective supports the call to move beyond simple “yes/no” judgments about non-standard media and instead quantify how particular formulations deviate from MHA for specific antibiotic-organism combinations before integrating them into AST workflows.

The composite ranking framework used in our study offers a pragmatic way to summarize the multi-dimensional performance and may be useful for other laboratories seeking to benchmark local media against MHA. In these analyses, BHI, LBA, and RSTA emerged as the closest overall surrogates, followed by NA, whereas PCA and PWA ranked lowest, indicating that not all “alternative” media are equal in their risk of introducing interpretive error. However, even the best-performing candidates fell short of full equivalence, and none were evaluated against clinical breakpoints or real patient isolates, which remain critical steps before changing routine practice.

The strain-media interaction patterns (Fig. 2) show that inhibition zones in disk diffusion are shaped by a combination of both medium formulation and organism-specific susceptibility, rather than by the agar type alone. MHA, as expected from its optimized composition and role as the CLSI/EUCAST reference medium, tends to produce larger zones across most strains, whereas other evaluated media were grouped according to their shared response patterns rather than by the simple “large versus small” zones. This supports previous work demonstrating that differences in nutrient content, ionic strength, and agar concentration can alter antibiotic diffusion and gradient shape, leading to medium-dependent zone sizes even when MICs remain stable (4, 20, 28, 31, 33, 34). The strain clustering is also biologically plausible as the Gram-positive reference strains, such as *Enterococcus faecalis, Bacillus subtilis,* and *Staphylococcus aureus* (except *B. cereus*), cluster together with uniformly higher inhibition, while *Klebsiella aerogenes* and *Escherichia coli* form separate clusters characterized by smaller or more variable zones, reflecting known intrinsic resistance traits and growth behaviors. Similar organism-dependent patterns have been reported when non-MHA media are used, with Gram-negative rods and spore-forming bacilli particularly sensitive to changes in agar composition or depth (4, 16, 28, 35). Collectively, these findings reinforce that performance deviations on non-standard media are both strain-dependent and medium-specific, which complicates the substitution of MHA in routine AST and risks inconsistent categorization of susceptibility results in resource-limited laboratories that rely on alternative media without formal validation.

The regression analysis (Fig. 3) in our study provides a quantitative view of how closely each evaluated medium aligns with MHA across multiple organism-antibiotic combinations, and this pattern aligns well with current understanding of media effects on disk-diffusion testing. MHA is formulated to standardize antibiotic diffusion and cation content; thus, high correlation with its zone diameters is a prerequisite for any candidate substitute. The strong linear associations observed for LBA and RSTA (r ≈ 0.83–0.79, R² ≈ 0.70–0.63) indicate that these media capture much of the variation seen on MHA and could, in principle, support reasonably predictable zone responses across diverse agents, similar to reports where enriched media produced susceptibility profiles broadly comparable to MHA for selected organisms (30). In contrast, NA, BHI, and PWA show only moderate correlations, and PCA performs poorly (r ≈ 0.51, R² ≈ 0.26), which parallels prior findings that nutrient or plate-count formulations generate more erratic diffusion behaviors and weaker agreement with reference media because of differences in agar concentration, nutrient density, and ionic strength (7, 20, 28). Equally important is the consistent finding that all regression slopes fall below the line of identity, showing that every alternative medium systematically underestimates zone diameters relative to MHA. Studies manipulating agar depth and composition within MHA itself have shown that even relatively small systematic shifts in zone size can move results across CLSI or EUCAST breakpoints, leading to major and very-major interpretive errors despite high correlation coefficients (4, 28, 35, 36). The negative bias observed in our study, most pronounced for PCA, therefore carries real risk, particularly for isolates whose true zone diameters lie close to clinical cut-offs. This combination of non-equivalent slopes, medium-specific scatter, and reduced R² supports the conclusion that correlation alone is not sufficient evidence of interchangeability; media can be highly correlated yet still yield biased or error-prone categorical interpretations if their regression lines deviate from the 1:1 relationship. Therefore, for laboratories in low- and middle-income countries, where non-standard media are often used out of necessity, these results provide concrete quantitative backing to concerns raised in reviews of clinical bacteriology practice (13, 15, 31, 33). This suggests that while LBA and RSTA may approximate MHA more closely than NA, PWA, or PCA, none of the evaluated media achieve the level of quantitative fidelity and proportional agreement expected for a true replacement. Without local validation and, potentially, medium-specific adjustment of interpretive criteria, the use of these media in the face of scarce resources could introduce systematic under-calling of susceptibility or over-calling of resistance in ways that are difficult to detect from routine quality-control data alone. Consequently, the regression findings reinforce the central message of our study that MHA should remain the preferred medium for disk-diffusion AST wherever feasible, and if any substitution in resource-limited settings, it must be guided by rigorous comparative data rather than by correlation metrics in isolation.

Our findings underscore that Mueller–Hinton agar remains the gold standard for AST, and deviations from this standard introduce interpretive errors. This study quantifies these errors to inform quality improvement and risk mitigation strategies in settings where access to standardized materials is limited.

### Limitations and future study directions

We acknowledge several limitations of this study. Firstly, the nine reference strains used here were ATCC/NCIMB laboratory reference organisms; their susceptibility profiles may not fully represent the phenotypic and genetic diversity of clinical isolates encountered in routine diagnostic settings. Categorical agreement and error rates observed in this study using reference strains should therefore be interpreted cautiously when extrapolating to clinical specimens, where isolates near breakpoint boundaries may be more common. Secondly, of the nine reference strains, only *Enterococcus faecalis* ATCC 29212 is a designated CLSI disk diffusion quality control organism; the remaining eight strains are characterized reference organisms to which formal CLSI QC pass/fail criteria may not be applied. Thirdly, only eight antibiotics spanning five drug classes were tested; the conclusions may not extend to other antibiotic classes with different molecular weights, protein binding, or diffusion kinetics. Furthermore, clarithromycin could not be included in categorical agreement analyses because there are no CLSI M100 disk diffusion breakpoints for the tested organisms, leaving its interpretive performance on alternative media uncharacterized. Future work should extend this approach to clinically derived isolates to determine whether the categorical agreement and error rates observed here are maintained in practice, explore medium-specific adjustment of zone interpretive criteria as a strategy to safely accommodate validated alternatives in resource-limited settings, and investigate whether the positive bias observed for ceftriaxone on several alternative media (PWA, BHI, PCA, RSTA) represents a reproducible finding with clinical implications. In the interim, the data presented here argue for continued prioritization of MHA procurement and quality control wherever feasible, and for cautious, evidence-based use of alternative agar formulations as a temporary resilience strategy rather than a wholesale replacement for the current global AST standard. Critically, a considerably larger validation study incorporating a sizeable and diverse cohort of clinical isolates representing varied resistance phenotypes and mechanisms is necessary to more robustly evaluate the performance of alternative AST media and to determine whether any such medium could serve as a viable substitute for MHA in resource-limited settings. The present proof-of-concept study, given its limited isolate panel (*n* = 9 reference strains), is not adequately powered to draw definitive conclusions about media equivalence or replacement.

### Conclusion

This study showed that while several routinely available culture media can partially approximate Mueller–Hinton agar, none fully reproduce its diffusion behavior, quantitative agreement, or ZDA across the nine reference strains and eight antibiotics tested. Media, such as BHI, LBA, and RSTA, were closest to MHA but still showed significant negative bias, sub-90% ZDA, and medium- and strain-specific deviations, whereas NA, PWA, and especially PCA performed less favorably. Categorical agreement, assessed for applicable organism-antibiotic combinations using CLSI M100 (2024) breakpoints, ranged from 92.3% (BHI, NA, PWA, PCA) to 96.2% (LBA and RSTA); no very major errors were detected, though major errors driven by ofloxacin against *Staphylococcus aureus* and minor errors involving amikacin underline the interpretive risk near breakpoint boundaries. These findings underscore that substituting MHA with non-standard media in resource-limited settings should occur after rigorous local validation and, where necessary, medium-specific adjustment of interpretive criteria, rather than assuming automatic equivalence based on convenience or availability.

## Data Availability

All data generated during this study are available within the article and its supplemental material. All individual zone diameter measurements (*n* = 1,512; nine bacterial strains × eight antibiotics × seven agar media × three replicates) are provided in full as Data Set S1, enabling independent verification of all summary statistics, coefficients of variation, categorical agreement calculations, and derived performance metrics reported in the article.

## SUPPLEMENTAL MATERIAL

Supplemental figures spectrum.04218-25-s0001.docx Fig. S1 to S6.

Supplemental tables spectrum.04218-25-s0002.docx Tables S1 and S2.

Data Set S1 spectrum.04218-25-s0003.csv Raw antibiotic susceptibility testing data set showing inhibition zone diameter measurements.

ASM does not own the copyrights to Supplemental Material that may be linked to, or accessed through, an article. The authors have granted ASM a non-exclusive, world-wide license to publish the Supplemental Material files. Please contact the corresponding author directly for reuse.
