# Supplementary material for: Beyond Mueller–Hinton agar: comparative evaluation of agar media for antibiotic susceptibility testing and implications for resource-limited laboratory settings
Source: Microbiol Spectr. 2026 May 29;14(7):e04218-25. doi: 10.1128/spectrum.04218-25 (PMC13340060; doi:10.1128/spectrum.04218-25)
Supplement: Supplemental figures — Fig. S1 to S6. [file spectrum.04218-25-s0001.docx]

Supplementary Figures


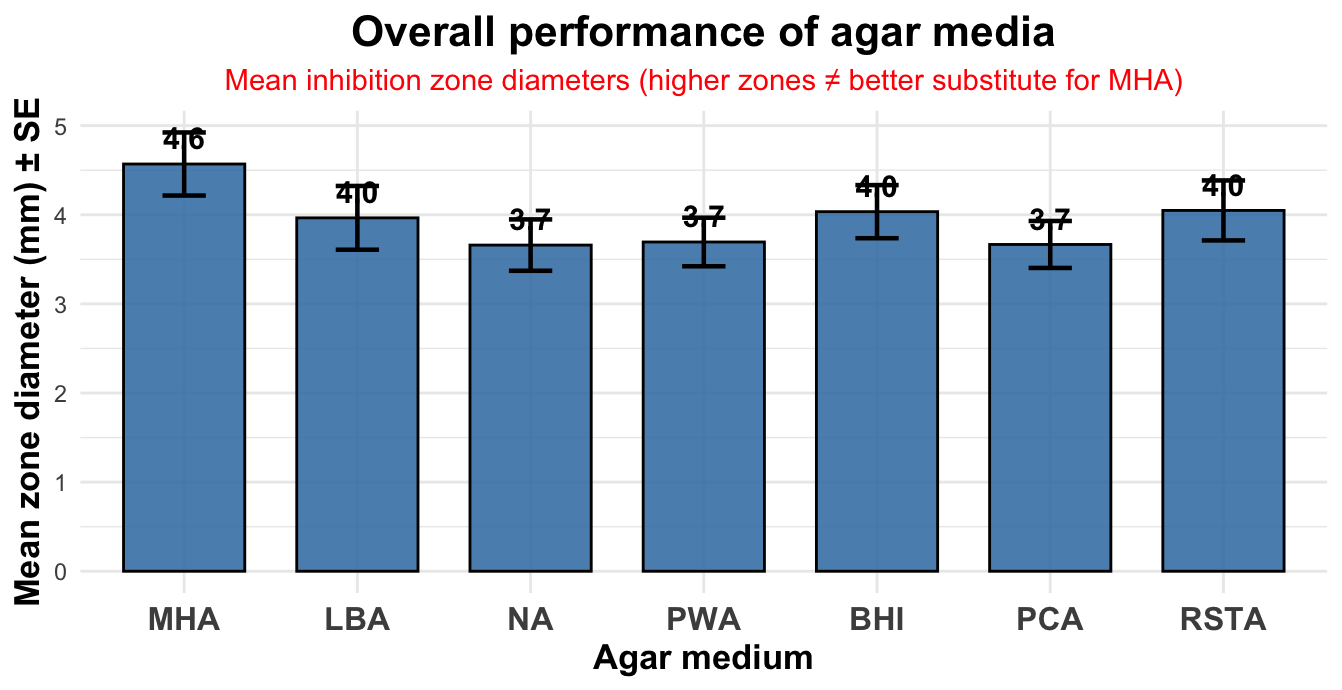


Supplementary Figure 1. Comparison of mean inhibition zone diameters across agar media relative to Mueller–Hinton agar (MHA).


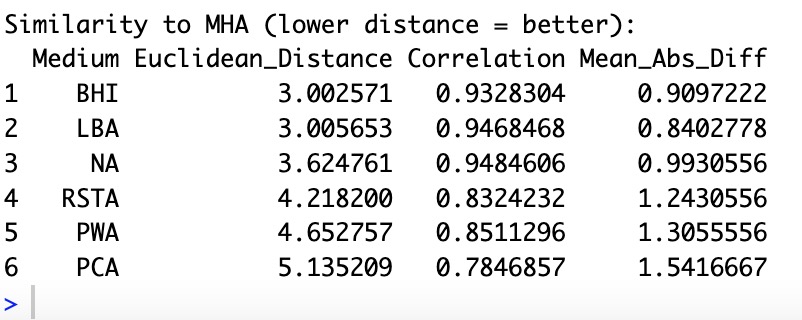


Supplementary Figure 2. Euclidean distance from MHA, quantifying how closely each medium approximates the inhibition profile of the standard

**
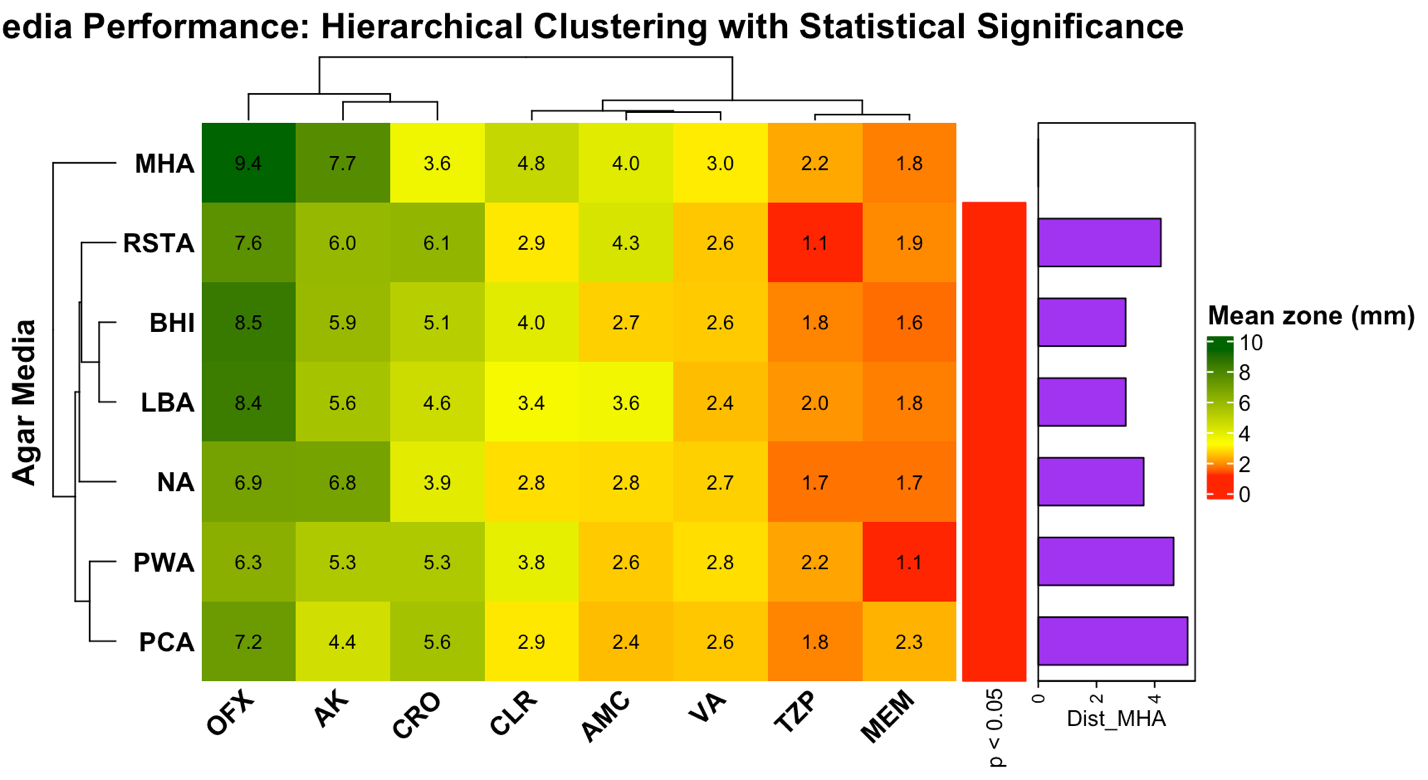
**

Supplementary Figure 3. Hierarchical clustering of agar media performance and statistical similarity to Mueller–Hinton agar (MHA) in antibiotic susceptibility testing

Key: Antibiotic abbreviation -OFX (ofloxacin), AMC (amoxicillin–clavulanate), AK (amikacin), CLR (clarithromycin), TZP (piperacillin–tazobactam), CRO (ceftriaxone), VA (vancomycin), and MEM (meropenem). Media abbreviations are already described in the methodology at their first appearance.


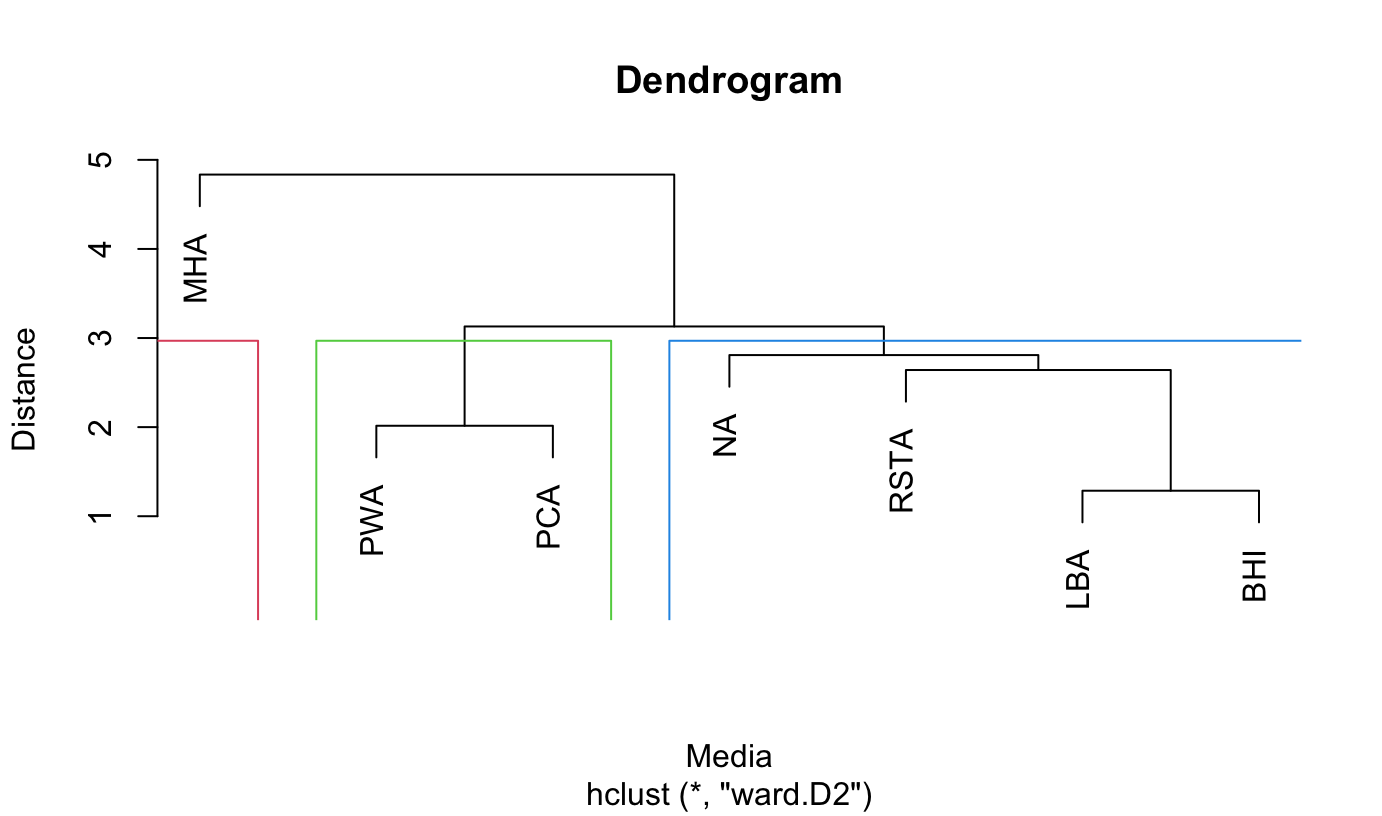


Supplementary Figure 4. Hierarchical clustering of agar media based on overall antibiotic inhibition profiles.


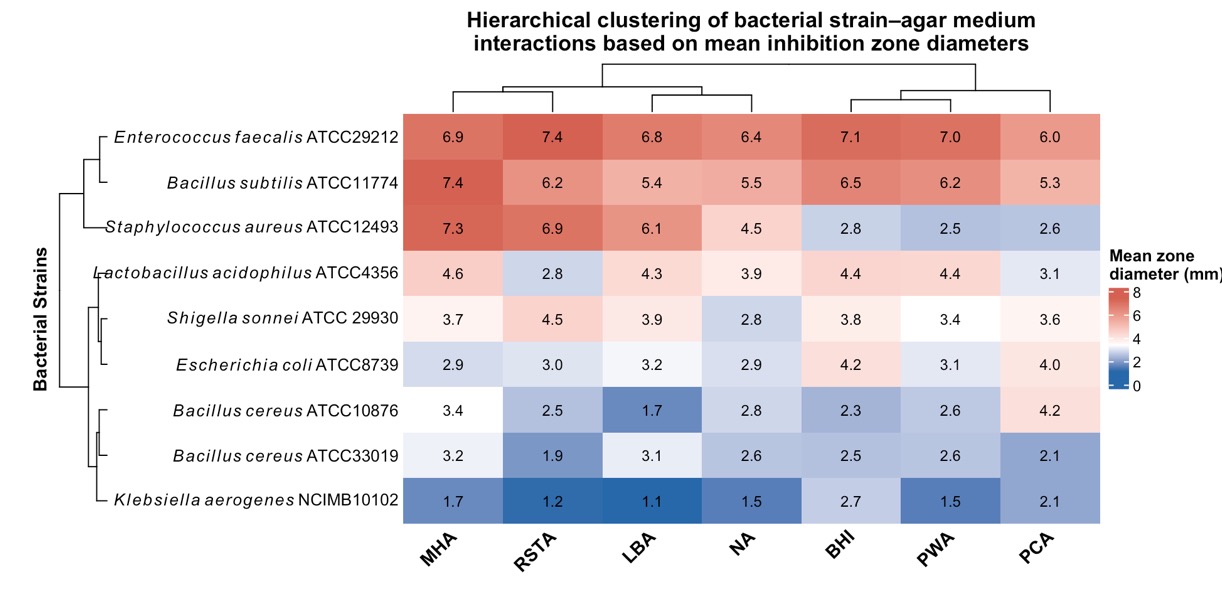


Supplementary Figure 5. Hierarchical clustering of bacterial strain–agar medium interactions based on mean inhibition zone diameters.

Key: Antibiotic abbreviation -OFX (ofloxacin), AMC (amoxicillin–clavulanate), AK (amikacin), CLR (clarithromycin), TZP (piperacillin–tazobactam), CRO (ceftriaxone), VA (vancomycin), and MEM (meropenem).


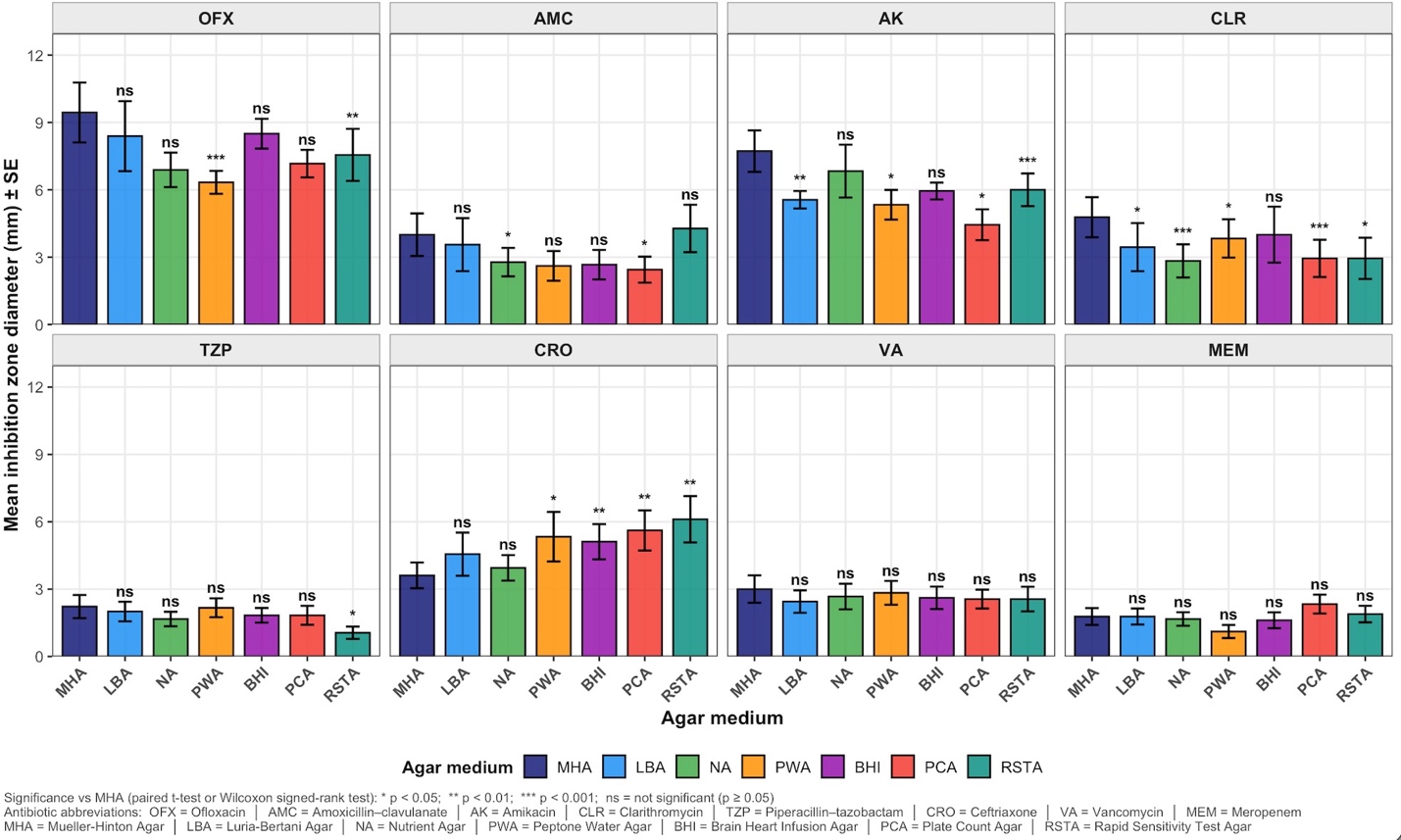


Supplementary Figure 6. Comparative performance of agar media across eight antibiotics based on mean inhibition zone diameters
