# Supplementary material for: Beyond Mueller–Hinton agar: comparative evaluation of agar media for antibiotic susceptibility testing and implications for resource-limited laboratory settings
Source: Microbiol Spectr. 2026 May 29;14(7):e04218-25. doi: 10.1128/spectrum.04218-25 (PMC13340060; doi:10.1128/spectrum.04218-25)
Supplement: Supplemental tables — Tables S1 and S2. [file spectrum.04218-25-s0002.docx]

**Supplementary Table 1.** Statistical output for pairwise comparisons of inhibition zone diameters on each alternative medium versus Mueller-Hinton agar (MHA) for each antibiotic (n = 27 pairs per comparison: 9 strains × 3 replicates).

| **Antibiotic** | **Medium vs MHA** | **n (pairs)** | **Mean MHA (mm)** | **Mean Alt. (mm)** | **Mean diff. (mm)** | **Test selected** | **Shapiro-Wilk p (normality)** | **t-statistic** | **df** | **t-test p-value** | **Wilcoxon statistic** | **Wilcoxon p-value** | **Chosen p-value** | **Sig.** |
| --- | --- | --- | --- | --- | --- | --- | --- | --- | --- | --- | --- | --- | --- | --- |
| **Ofloxacin (OFX)** | LBA | 27 | 9.44 | 8.44 | -1.00 | Wilcoxon | <0.001 | −1.665 | 26 | 0.1089 | 38.0 | 0.0431 | **0.0431** | ***** |
| Ofloxacin (OFX) | NA | 27 | 9.44 | 7.22 | -2.22 | Wilcoxon | <0.001 | −1.907 | 26 | 0.0673 | 47.5 | 0.0131 | **0.0131** | ***** |
| Ofloxacin (OFX) | PWA | 27 | 9.44 | 6.67 | -2.78 | Wilcoxon | <0.001 | −2.830 | 26 | 0.0087 | 4.0 | 0.0000 | **0.0000** | ******* |
| Ofloxacin (OFX) | BHI | 27 | 9.44 | 8.89 | -0.56 | Wilcoxon | <0.001 | −0.680 | 26 | 0.5026 | 72.0 | 0.5460 | **0.5460** | **ns** |
| Ofloxacin (OFX) | PCA | 27 | 9.44 | 7.44 | -2.00 | Wilcoxon | <0.001 | −1.489 | 26 | 0.1491 | 64.0 | 0.2376 | **0.2376** | **ns** |
| Ofloxacin (OFX) | RSTA | 27 | 9.44 | 7.67 | -1.78 | Paired t-test | 0.3150 | −3.637 | 26 | 0.0012 | 13.0 | 0.0007 | **0.0007** | ******* |
| **Amoxicillin–clav. (AMC)** | LBA | 27 | 4.00 | 3.59 | -0.41 | Wilcoxon | <0.001 | −0.792 | 26 | 0.4358 | 28.0 | 0.1114 | **0.1114** | **ns** |
| Amoxicillin–clav. (AMC) | NA | 27 | 4.00 | 2.85 | -1.15 | Wilcoxon | <0.001 | −2.013 | 26 | 0.0547 | 4.5 | 0.0088 | **0.0088** | ****** |
| Amoxicillin–clav. (AMC) | PWA | 27 | 4.00 | 2.74 | -1.26 | Wilcoxon | <0.001 | −1.840 | 26 | 0.0775 | 19.0 | 0.0335 | **0.0335** | ***** |
| Amoxicillin–clav. (AMC) | BHI | 27 | 4.00 | 2.81 | -1.19 | Wilcoxon | <0.001 | −1.856 | 26 | 0.0749 | 14.0 | 0.0401 | **0.0401** | ***** |
| Amoxicillin–clav. (AMC) | PCA | 27 | 4.00 | 2.59 | -1.41 | Paired t-test | 0.2609 | −2.530 | 26 | 0.0178 | 17.5 | 0.0143 | **0.0143** | ***** |
| Amoxicillin–clav. (AMC) | RSTA | 27 | 4.00 | 4.19 | +0.19 | Wilcoxon | <0.001 | 0.349 | 26 | 0.7296 | 56.5 | 0.9352 | **0.9352** | **ns** |
| **Amikacin (AK)** | LBA | 27 | 7.72 | 5.59 | -2.13 | Wilcoxon | <0.001 | −2.790 | 26 | 0.0097 | 11.0 | 0.0006 | **0.0006** | ******* |
| Amikacin (AK) | NA | 27 | 7.72 | 6.85 | -0.87 | Paired t-test | 0.5930 | −2.773 | 26 | 0.0101 | 25.0 | 0.0087 | **0.0087** | ****** |
| Amikacin (AK) | PWA | 27 | 7.72 | 5.44 | -2.28 | Wilcoxon | <0.001 | −2.542 | 26 | 0.0172 | 13.5 | 0.0014 | **0.0014** | ****** |
| Amikacin (AK) | BHI | 27 | 7.72 | 6.07 | -1.65 | Wilcoxon | <0.001 | −1.786 | 26 | 0.0857 | 29.5 | 0.0421 | **0.0421** | ***** |
| Amikacin (AK) | PCA | 27 | 7.72 | 4.56 | -3.16 | Wilcoxon | <0.001 | −2.535 | 26 | 0.0176 | 24.5 | 0.0032 | **0.0032** | ****** |
| Amikacin (AK) | RSTA | 27 | 7.72 | 6.00 | -1.72 | Wilcoxon | <0.001 | −4.846 | 26 | 0.0001 | 3.5 | 0.0001 | **0.0001** | ******* |
| **Clarithromycin (CLR)** | LBA | 27 | 4.78 | 3.48 | -1.30 | Wilcoxon | <0.001 | −2.147 | 26 | 0.0414 | 27.0 | 0.0180 | **0.0180** | ***** |
| Clarithromycin (CLR) | NA | 27 | 4.78 | 2.85 | -1.93 | Wilcoxon | <0.001 | −4.127 | 26 | 0.0003 | 7.5 | 0.0000 | **0.0000** | ******* |
| Clarithromycin (CLR) | PWA | 27 | 4.78 | 3.85 | -0.93 | Wilcoxon | <0.001 | −2.718 | 26 | 0.0116 | 14.5 | 0.0037 | **0.0037** | ****** |
| Clarithromycin (CLR) | BHI | 27 | 4.78 | 4.07 | -0.71 | Wilcoxon | <0.001 | −1.028 | 26 | 0.3132 | 31.5 | 0.0270 | **0.0270** | ***** |
| Clarithromycin (CLR) | PCA | 27 | 4.78 | 2.96 | -1.82 | Paired t-test | 0.2324 | −5.054 | 26 | 0.0000 | 4.5 | 0.0000 | **0.0000** | ******* |
| Clarithromycin (CLR) | RSTA | 27 | 4.78 | 2.96 | -1.82 | Wilcoxon | <0.001 | −2.668 | 26 | 0.0132 | 25.0 | 0.0090 | **0.0090** | ****** |
| **Piperacillin–taz. (TZP)** | LBA | 27 | 2.22 | 2.07 | -0.15 | Wilcoxon | <0.001 | −0.468 | 26 | 0.6437 | 35.0 | 0.8409 | **0.8409** | **ns** |
| Piperacillin–taz. (TZP) | NA | 27 | 2.22 | 1.74 | -0.48 | Wilcoxon | <0.001 | −1.567 | 26 | 0.1296 | 26.5 | 0.0475 | **0.0475** | ***** |
| Piperacillin–taz. (TZP) | PWA | 27 | 2.22 | 2.19 | -0.04 | Paired t-test | 0.0703 | −0.181 | 26 | 0.8578 | 38.5 | 0.9030 | **0.9030** | **ns** |
| Piperacillin–taz. (TZP) | BHI | 27 | 2.22 | 1.89 | -0.33 | Paired t-test | 0.3100 | −0.961 | 26 | 0.3454 | 29.5 | 0.3500 | **0.3500** | **ns** |
| Piperacillin–taz. (TZP) | PCA | 27 | 2.22 | 1.89 | -0.33 | Paired t-test | 0.4587 | −0.961 | 26 | 0.3454 | 42.5 | 0.3052 | **0.3052** | **ns** |
| Piperacillin–taz. (TZP) | RSTA | 27 | 2.22 | 1.22 | -1.00 | Paired t-test | 0.3543 | −3.162 | 26 | 0.0040 | 22.0 | 0.0056 | **0.0040** | ****** |
| **Ceftriaxone (CRO)** | LBA | 27 | 3.61 | 4.56 | +0.94 | Wilcoxon | <0.001 | 1.568 | 26 | 0.1293 | 24.0 | 0.0802 | **0.0802** | **ns** |
| Ceftriaxone (CRO) | NA | 27 | 3.61 | 3.93 | +0.31 | Paired t-test | 0.3635 | 1.065 | 26 | 0.2960 | 28.0 | 0.1946 | **0.1946** | **ns** |
| Ceftriaxone (CRO) | PWA | 27 | 3.61 | 5.26 | +1.64 | Wilcoxon | <0.001 | 2.480 | 26 | 0.0198 | 8.0 | 0.0036 | **0.0036** | ****** |
| Ceftriaxone (CRO) | BHI | 27 | 3.61 | 5.04 | +1.44 | Wilcoxon | <0.001 | 4.603 | 26 | 0.0001 | 0.5 | 0.0001 | **0.0001** | ******* |
| Ceftriaxone (CRO) | PCA | 27 | 3.61 | 5.56 | +1.94 | Wilcoxon | <0.001 | 3.431 | 26 | 0.0019 | 1.0 | 0.0003 | **0.0003** | ******* |
| Ceftriaxone (CRO) | RSTA | 27 | 3.61 | 6.07 | +2.46 | Wilcoxon | <0.001 | 3.858 | 26 | 0.0007 | 8.0 | 0.0002 | **0.0002** | ******* |
| **Vancomycin (VA)** | LBA | 27 | 3.00 | 2.48 | -0.52 | Wilcoxon | <0.001 | −1.890 | 26 | 0.0697 | 9.5 | 0.0222 | **0.0222** | ***** |
| Vancomycin (VA) | NA | 27 | 3.00 | 2.67 | -0.33 | Wilcoxon | <0.001 | −2.062 | 26 | 0.0493 | 4.0 | 0.0129 | **0.0129** | ***** |
| Vancomycin (VA) | PWA | 27 | 3.00 | 2.85 | -0.15 | Wilcoxon | <0.001 | −0.825 | 26 | 0.4165 | 23.5 | 0.2442 | **0.2442** | **ns** |
| Vancomycin (VA) | BHI | 27 | 3.00 | 2.63 | -0.37 | Wilcoxon | <0.001 | −2.122 | 26 | 0.0435 | 4.0 | 0.0146 | **0.0146** | ***** |
| Vancomycin (VA) | PCA | 27 | 3.00 | 2.59 | -0.41 | Wilcoxon | <0.001 | −1.288 | 26 | 0.2089 | 28.5 | 0.0647 | **0.0647** | **ns** |
| Vancomycin (VA) | RSTA | 27 | 3.00 | 2.59 | -0.41 | Wilcoxon | <0.001 | −1.512 | 26 | 0.1428 | 13.5 | 0.0531 | **0.0531** | **ns** |
| **Meropenem (MEM)** | LBA | 27 | 1.78 | 1.78 | 0.00 | Wilcoxon | <0.001 | 0.000 | 26 | 1.0000 | 20.5 | 0.9364 | **0.9364** | **ns** |
| Meropenem (MEM) | NA | 27 | 1.78 | 1.67 | -0.11 | Wilcoxon | <0.001 | −0.256 | 26 | 0.8003 | 42.5 | 0.8390 | **0.8390** | **ns** |
| Meropenem (MEM) | PWA | 27 | 1.78 | 1.19 | -0.59 | Wilcoxon | <0.001 | −1.844 | 26 | 0.0769 | 20.5 | 0.0358 | **0.0358** | ***** |
| Meropenem (MEM) | BHI | 27 | 1.78 | 1.67 | -0.11 | Wilcoxon | <0.001 | −0.437 | 26 | 0.6656 | 24.5 | 0.5261 | **0.5261** | **ns** |
| Meropenem (MEM) | PCA | 27 | 1.78 | 2.26 | +0.48 | Wilcoxon | <0.001 | 1.890 | 26 | 0.0697 | 3.5 | 0.0166 | **0.0166** | ***** |
| Meropenem (MEM) | RSTA | 27 | 1.78 | 1.89 | +0.11 | Wilcoxon | <0.001 | 0.461 | 26 | 0.6489 | 14.5 | 0.5773 | **0.5773** | **ns** |

*Test selection: Shapiro-Wilk test applied to pairwise differences; Paired t-test used where p > 0.05 (normality supported); Wilcoxon signed-rank test used where p ≤ 0.05. Significance: *** p < 0.001; ** p < 0.01; * p < 0.05; ns = not significant (p ≥ 0.05). Positive mean differences (green) indicate the alternative medium produced larger zones than MHA; negative (red) indicate smaller zones. Both test statistics are reported for transparency. Abbreviations — Media: MHA = Mueller-Hinton Agar; LBA = Luria-Bertani Agar; NA = Nutrient Agar; PWA = Peptone Water Agar; BHI = Brain Heart Infusion Agar; PCA = Plate Count Agar; RSTA = Rapid Sensitivity Test Agar.*

Supplementary Table 2. Summary of similarity, agreement, statistical tests, reproducibility, and bias for all agar media relative to MHA.

| **Medium** | **Euclid Dist** | **Corr (r)** | **Mean Abs Diff** | **N Pairs** | **Mean Diff** | **Cohen’s d** | **t-p value** | **Wilcoxon p** | **Bonferroni Sig** | **EA (%)** | **CV (%)** | **Mean Bias** |
| --- | --- | --- | --- | --- | --- | --- | --- | --- | --- | --- | --- | --- |
| **BHI** | 3 | 0.933 | 0.91 | 144 | –0.53 | –0.165 | 0.0499 | 0.136 | No | 77.1 | 8.22 | –0.53 |
| **LBA** | 3.01 | 0.947 | 0.84 | 144 | –0.60 | –0.245 | 0.0038 | 0.002 | Yes | 76.4 | 13.6 | –0.60 |
| **NA** | 3.62 | 0.948 | 0.99 | 144 | –0.91 | –0.334 | 0.0001 | 9.80E-06 | Yes | **83.3** | **5.32** | –0.91 |
| **RSTA** | 4.22 | 0.832 | 1.24 | 144 | –0.52 | –0.194 | 0.0213 | 0.005 | No | 71.5 | 11.1 | –0.52 |
| **PWA** | 4.65 | 0.851 | 1.31 | 144 | –0.88 | –0.284 | 0.00086 | 8.70E-05 | Yes | 81.9 | **15** | –0.88 |
| **PCA** | **5.14** | **0.785** | **1.54** | 144 | –0.90 | –0.239 | 0.0047 | 0.0089 | Yes | **69.4** | 12.4 | –0.90 |
